# Occupation of a thermoresistant-scaffold (αRep) at SP1-NC cleavage site disturbs the function of HIV-1 protease

**DOI:** 10.1042/BSR20201131

**Published:** 2020-06-23

**Authors:** Sudarat Hadpech, Nichakan Peerakam, Koollawat Chupradit, Chatchai Tayapiwatana

**Affiliations:** 1 Faculty of Pharmaceutical Sciences, Burapha University, Chon Buri 20131, Thailand; 2 Division of Clinical Immunology, Department of Medical Technology, Faculty of Associated Medical Sciences, Chiang Mai University, Chiang Mai 50200, Thailand; 3 Center of Biomolecular Therapy and Diagnostic, Faculty of Associated Medical Sciences, Chiang Mai University, Chiang Mai 50200, Thailand; 4 Biomedical Technology Research Center, National Center for Genetic Engineering and Biotechnology, National Science and Technology Development Agency at The Faculty of Associated Medical Sciences, Chiang Mai University, Chiang Mai, Thailand

**Keywords:** αRep, HIV-1 nucleocapsid, HIV-1 therapy, HIV-1, nucleocapsid inhibitor, scaffold protein

## Abstract

HIV-1 nucleocapsid (NC) becomes an attractive target for the development of novel anti-HIV-1 agents. Discovering of non-antibody scaffolds that disrupt the function of NC will be a potential aspect for disturbing viral maturation process. Correspondingly, we explored the specific binding site of the thermoresistant-scaffold protein, αRep9A8 which formerly demonstrated the inhibitory effect on HIV-1 replication. The portion of Gag, CA_21_-SP1-NC has been used as a template for designing nine overlapping peptides (P4–P12). The P9 peptide showed the strongest binding activity followed by P8 and P12 respectively. The amino acid sequences on those peptides resemble the N-terminal domain of the NC proximity to the SP1-NC initial cleavage site and across the conserved CCHC zinc finger 1 (ZF1) of NC. The interaction *K*_D_ between αRep9A8 with its target was 224.9 ± 57.4 nM. Consequently, αRep9A8 demonstrated the interference of the HIV-1 protease function by hindering a protease cleavage site. The released NC product from CA_21_-SP1-NC was diminished. The present study provided an additional information of αRep9A8 function in interfering of viral maturation processes resulting in the decremental efficiency of viral infectivity.

## Introduction

HIV-1 particle egresses from the plasma membrane of infected cells in an immature particle. Gag polyprotein is the major component of the viral progeny which requires the proteolytic cleavage to become a mature infectious virus [1]. Maturation process changes the virion morphology from the donut-shaped particle to the conical shaped. Inside the particle is lined with viral matrix proteins (MA), in the middle, containing a condensed cone-shaped core composed of a viral capsid (CA) which finally encapsidates the ribonucleoprotein (RNP) complex, comprising viral genomic RNA, nucleocapsid (NC), and viral enzymes [2]. Among the five proteolytic cleavage sites on Gag, the rate of cleaving by HIV-1 protease enzyme are different. The fastest cleaving occurs at the SP1–NC junction. Relative to this event, cleaving at SP2-p6 is 9× slower, MA-CA is 14× slower, NC-SP2 is 350× slower, and CA-SP1 is 400× slower [1–3]. The proteolytic process allows four major domains of Gag; MA, CA, NC, and p6 to be free and perform its functions.

HIV-1 NC is highly conserved in the amino acid sequences among diverse HIV-1 subtypes and is necessary for a large spectrum of virus activities. NC is a nucleic acid-binding domain and plays a key role in chaperoning activities. The two zinc finger motifs within NC, zinc finger 1 (ZF1), and ZF2 are necessary to interact with the Psi (ψ) packaging signal of viral gRNA during viral assembly. The chaperone activities on HIV-1 RNA and DNA within the viral particle as well as during the reverse transcription step are also exerted by NC. There are 55 amino acids in the NC domain, each zinc finger motif contains two strictly conserved CCHC which act as a Zn^2+^ binding residue [4]. The studies showed that a point mutation within the zinc-binding motifs leading to losing infectivity of the virus [5–8]. Considering its highly conserved amino acid sequence among clades and participation in the crucial steps of viral replication, NC becomes an attractive target for the development of HIV-1 universal inhibitors [4,9]. Zinc ejector was initially developed in 1993 by Rice et al. [10]. The mechanisms of zinc ejector anti-NC compounds can be classified into three groups; electrophilic attack of the zinc fingers, zinc ejection through chelation, and covalent binding of Cys residues by platinum [4]. In addition to zinc ejectors, non-covalent NC inhibitors (NCIs) have also been reported and classified. The first group is non-covalent NCIs binding to NC exhibiting the inhibitory effect on the chaperoning activity [11]. The second group is the molecules that promote the destabilization of the secondary structure of nucleic acid interfering with viral genome selection and packaging efficiency [11].

Currently, non-antibody protein scaffolds are now translating from research laboratory into clinical development. FDA recently approved abicipar pegol (Allergan) (MP0112), a designed ankyrin repeat protein (DARPin) binding to and inhibiting the biologic activity of human vascular endothelial growth factor (VEGF) A with high affinity and specificity [12–14]. Several protein scaffolds have been reported which should be an alternative for therapy [12]. Another type of artificial protein derived from a natural family of thermoresistant α-helical repeat (αRep protein) became well-known recently [15,16]. Its biophysical properties are highly favorable to medical applications. The αRep and DARPin share many common advantages: disulfide independent folding, high solubility, active inside living cells and also extracellular compartment [16–18], and interact to target with high binding affinity [19].

In the present study, we investigated the specific binding site of αRep9A8 on the HIV-1 NC and its mechanism of actions against the HIV-1 replication cycle. The scaffold protein αRep9A8 previously reported as HIV-1 inhibitors in cell-based assay, however, the mode of action has not been clarified [17]. Identification of the key amino acids, determination of binding affinity, and elucidating anti-HIV-1 PR activity of αRep9A8 will provide a piece of additional molecular information for future applications.

## Materials and methods

### Expression and purification of αRep protein

The *E. coli* M15[pREP4] harboring the pQE31-αRep9A8 plasmid was grown in TR broth supplemented with ampicillin (100 μg/ml), kanamycin (25 μg/ml), and 1% (w/v) d-glucose with shaking at 37°C. When the OD_600_ reached 0.8–1, the αRep9A8 expression was induced with 1 mM PITG, and the culture was further incubated for 16 h at 30°C with shaking. Bacterial cells were pelleted by centrifugation (1200×***g*** for 30 min at 4°C), resuspended in PBS containing a cocktail of protease inhibitors (PIs) (Roche Diagnostics GmbH), and lysed cells by sonication. Bacterial cell lysates were clarified by centrifugation at 15000×***g*** for 30 min at 4°C. Soluble αRep9A8 was then purified by affinity chromatography on HisTrap columns (GE Healthcare Life Sciences), and analyzed by SDS/PAGE and Western blotting.

### Expression of GST-CA_21_-SP1-NC

The BL21 cells were used for the production of recombinant Glutathione-S-transferase (GST)-CA_21_-SP1-NC. The BL21 harboring pGEX-GST-CA_21_-SP1-NC was cultured in TR broth supplemented with ampicillin (100 μg/ml), kanamycin (25 μg/ml), and 1% (w/v) d-glucose with shaking at 37°C. A total of 1 mM IPTG was then added to the culture when the OD_600_ reached 0.8–1. The culture was then further incubated at 30°C with shaking for 16 h. Bacteria were pelleted by centrifugation (1200×***g*** for 30 min at 4°C), resuspended in PBS containing a cocktail of PIs (Roche Diagnostics GmbH), and lysed cells by sonication. Bacterial cell lysates were clarified by centrifugation at 15000×***g*** for 30 min at 4°C and analyzed by SDS/PAGE and Western blotting.

### Expression of HIV-PRH_6_

The plasmid pET21-HIV-PRH_6_ was transformed into *E. coli* strain BL21(DE3) as previously described by Kitidee et al. [20] Briefly, the bacterial cells were grown in TR broth supplemented with ampicillin (100 μg/ml), kanamycin (25 μg/ml), and 1% (w/v) d-glucose with shaking at 37°C with shaking until the OD_600_ reached 0.8–1. Subsequently, 0.1 mM IPTG was added to induce the production of the protein. The culture was shaken continuously at 18°C for 18 h. The cells were then pelleted by centrifugation (1200×***g*** for 30 min at 4°C) and resuspended in TBS pH 7.4. The cells were lyzed by ultrasonication and centrifuged at 15000×***g*** for 30 min at 4°C. The soluble fraction was collected and further analyzed by Western blotting using an anti-His tag monoclonal antibody.

### SDS/PAGE and Western blotting

Protein samples were separated by electrophoresis in SDS-containing 12% polyacrylamide gel. When the separation was done, gels were stained with PageBlue™ protein staining solution (Thermo Fisher Scientific) or used for Western blotting. In Western blotting, protein separated on the SDS gels were transferred to PVDF membrane (GE Healthcare, U.K.) then blocked with 5% skim milk in PBS. Subsequently, the membrane was incubated with primary antibodies and secondary antibodies. Mouse monoclonal anti-His_6_ (ABM) was used to detect αRep proteins and HIV-PRH_6_. GST-tagged protein was detected using an anti-GST antibody (ABM). Horseradish peroxidase (HRP)-conjugated secondary antibody was added after washed out the primary antibodies. The membrane substrate chromogen was added and the protein bands on the blot were scanned.

### Synthetic overlapping peptide design

**Figure 1:**
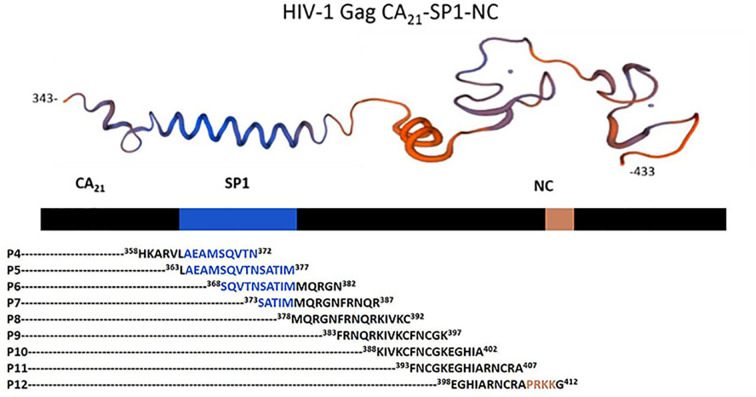
The 3D structure of the CA21-SP1-NC domain of HIV-1 Gag^343–433^ The protein model (*top panel*) generated from https://swissmodel.expasy.org. The amino acid sequence of short synthetic overlapping peptides (P4–P12) indicated with the position number.

In the previous study, we obtained αRep9A8 which specifically bound to the HIV-1 Gag polyprotein at the domain started from Gag343 to Gag433 so-called CA_21_-SP1-NC. Since this domain is 90 amino acids long, short synthetic overlapping peptides are needed to specify which region is exactly an epitope of αRep9A8. The synthetic peptides were designed by using the CA_21_-SP1-NC as a template to have 15 amino acids in length together with 10 amino acids overlapped sequence as indicated in Figure 1 and Table 1. The nine candidate synthetic overlapping peptides started from the C-terminal of CA to the ZF1 at ^358^HKAR–PRKKG^412^ ordered from Prima Scientific Co., Ltd at 1 mg, >95% purity. The peptides were prepared to obtain 1 mg/ml stock in PBS.

**Table 1:** Synthetic peptide sequences

Peptide name	Peptide sequence
P4	^358^HKARVLAEAMSQVTN^372^
P5	^363^LAEAMSQVTNSATIM^377^
P6	^368^SQVTNSATIMMQRGN^382^
P7	^373^SATIMMQRGNFRNQR^387^
P8	^378^MQRGNFRNQRKIVKC^392^
P9	^383^FRNQRKIVKCFNCGK^397^
P10	^388^KIVKCFNCGKEGHIA^402^
P11	^393^FNCGKEGHIARNCRA^407^
P12	^398^EGHIARNCRAPRKKG^412^

### Epitope mapping analysis by ELISA

The indirect ELISA was used to identify the binding site of the αRep9A8 on the CA_21_-SP1-NC target domain on the HIV-1 Gag polyprotein. The microtiter plate was coated with 50 μl of 10-mer overlapping synthetic peptides, diluted at 10 μg/ml in coating buffer (1 M NaHCO_3_, pH 9.3). Each peptide is 15 amino acids long. The coated microtiter plate was left overnight in a moist chamber at 4°C. Wells were washed three times with 0.05% Tween 20 in PBS and blocked the plate with blocking solution (1% skim milk in PBS) 200 μl/well at RT in a moist chamber for 1 h to prevent non-specific binding. Next, washed three times, followed by adding 100 μl/well of αRep9A8 diluted at 10 μg/ml for final concentration in blocking solution. Incubated the reaction at RT in a moist chamber for 1 h. The binding of αRep9A8 to each overlapping synthetic peptides was revealed by incubation with an HRP-conjugated anti-His tag antibody at RT for 1 h, followed by addition of 100 μl of TMB microwell peroxidase substrate. The 1 M HCl was used to stop the reaction, and OD measured at 450 nm.

The competitive ELISA was performed to confirm the binding reaction between αRep9A8 and target GST-CA_21_-SP1-NC. The microtiter plate was coated with 50 μl of 10 μg/ml of synthetic peptides diluted in coating buffer. The coated Microtiter plate was left overnight in a moist chamber at 4°C. Wells were washed three times with 0.05% Tween 20 in PBS and blocked the plate with blocking solution (1% skim milk in PBS) 200 μl/well at RT in a moist chamber for 1 h to prevent non-specific binding. The crude protein of target GST-CA_21_-SP1-NC at 0, 5, 10, and 20 μg/ml were pre-incubated with 20 μg/ml of αRep9A8 at RT with shaking for 1 h subsequently, the mixtures were added into the coated wells. The competitive reaction was revealed by incubation with an HRP-conjugated anti-His tag antibody at RT for 1 h, followed by the addition of 100 μl of TMB microwell peroxidase substrate. The 1 M HCl was used to stop the reaction, and OD measured at 450 nm.

### Binding kinetic analysis by bio-layer interferometry

The binding kinetics of αRep9A8, with target GST-CA_21_-SP1-NC, was measured using bio-layer interferometry (BLI) performed with the BLItz™ system (ForteBio, Menlo Park, CA). All interaction analyses were diluted in sample diluent (bovine serum albumin (w/v, 2%) and tween-20 detergent (v/v, 0.05%) in PBS). Anti-Penta-His (HIS1K) biosensors were hydrated for 10 min in sample diluent immediately before use. The purified αRep9A8, at 20 μg/ml was immobilized to Anti-Penta-His biosensor for 2 min. The biosensors were washed with sample diluent for 30 s and transferred to the tube containing 10 μg/ml of target GST-CA_21_-SP1-NC. The association and dissociation values of αRep9A8 were determined in each step. Kinetic parameters (*k_on_* and *k_off_*) and the equilibrium dissociation constant (*K*_D_) was calculated from a non-linear local fit of the data between αRep9A8 and its target GST-CA_21_-SP-NC by the BLItz Pro 1.1 software.

### Anti-HIV-1 proteolytic cleavage activity analysis of αRep9A8 by Western blotting

The anti-proteolytic cleavage activity of αRep9A8 was assessed by Western blotting analysis. The 20 µl of viral target protein GST-CA_21_-SP1-NC at 5 mg/ml was pre-incubated with 20 µl of αRep9A8 started at 2000, 1000, 500, 250, 125, and 62.5 µg/ml for 1 h at RT with shaking. The 20 µl of 5 mg/ml HIV-PRH_6_ was added to the mixture reaction and further incubated for 1 h. The mixtures were then analyzed the anti-PR activity by Western blotting. The ratio of the reaction mixture is GST-CA_21_-SP1-NC: αRep9A8: HIV-PRH_6_ = 20 µl: 20 µl: 20 µl. The cleaved and non-cleaved products were revealed by anti-GST antibody (ABM) and HRP–conjugated secondary antibody. The membrane substrate chromogen was added and the protein bands on the blot were scanned.

## Results

### Preparation of recombinant proteins

**Figure 2:**
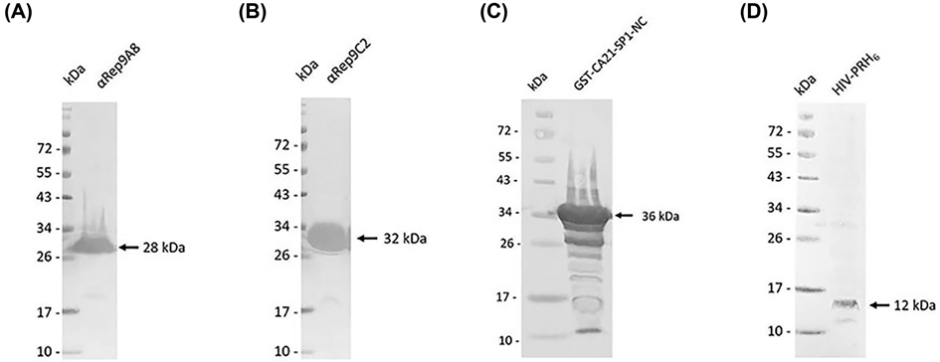
Western blotting analysis of recombinant protein preparation (**A,B**) Are His-tagged αRep proteins. αRep9A8 has a molecular mass of 28 kDa. αRep9C2, irrelevant control has a molecular mass of 32 kDa, (**C**) 36 kDa of GST-CA_21_-SP1-NC, (**D**) 12 kDa of HIV-PRH_6_.

The *E. coli* M15[pREP4] harboring the pQE31-αRep9A8 plasmid was used to express the His-tagged recombinant αRep9A8 protein. After purification by affinity chromatography using Ni^2+^ column, the protein fractions were assayed by SDS/PAGE and gel was stained by PageBlue™ protein staining solution. The results demonstrated that αRep9A8 has a molecular mass of 28 kDa (data not shown). The specific band of αRep9A8 at the same molecular weight was observed using anti-His tag monoclonal antibody in Western blotting analysis. Same as αRep9A8, the irrelevant control protein, αRep9C2 was obtained from the same steps of expression, purification, and analysis. The result showed that the protein band was observed at 32 kDa in both SDA/PAGE (data not shown) and Western blotting analysis (Figure 2A,B).

The viral target CA_21_-SP1-NC, spanned from L343 to F433 of the HIV-1 Gag polyprotein was constructed into pGEX plasmid by fused to GST gene as described previously [17]. The target protein was expressed in *E. coli* BL21. Crude protein after harvested was analyzed by Western blotting. The specific band at 36 kDa was observed when probed with anti-GST monoclonal antibody and HRP-conjugated secondary antibody (Figure 2C).

The HIV-PRH_6_ was produced in *E. coli* strain BL21(DE3) harboring plasmid pET21-HIV-PRH_6._ Bacterial cells were harvested and assayed for the HIV-PRH_6_ expression by Western blotting. The HIV-PRH_6_ protein band was observed at 12 kDa using anti-His tag monoclonal antibody (Figure 2D).

### Determination of αRep9A8 recognition site on CA_21_-SP1-NC

**Figure 3:**
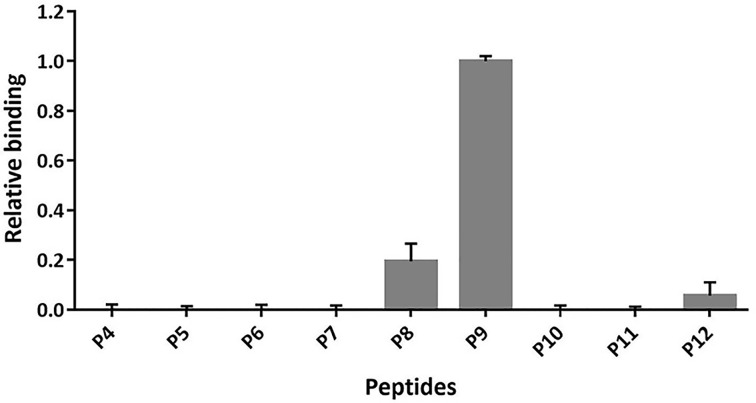
Determining the recognition site of αRep9A8 on viral target protein CA_21_-SP1-NC Indirect ELISA was used to investigate the interaction of αRep9A8 on immobilized synthetic peptides. Binding activities were reviewed by anti-His HPR and measured OD at 450 nm and the highest signal obtained from P9 was attributed the 1-unit value. *y-axis*; the relative biding activity, *x-axis*; list of peptides coated wells (P4–P12). Data shown represent the mean ± S.D. of the result in triplicates.

To identify the recognition site of αRep9A8 on viral target protein CA_21_-SP1-NC, indirect ELISA was performed by pre-coated short synthetic overlapping peptides to the microtiter wells. Binding of the αRep9A8 to the immobilized short synthetic overlapping peptides was reviewed by anti-His-HRP. The result demonstrated that αRep9A8 strongly interacted with P9 and the lower binding activity was observed from the wells immobilized P8 and P12, respectively. No interaction signal was observed from the rest of the short synthetic overlapping peptides, P4–P7 and P10–P11 (Figure 3) and the αRep9C2 irrelevant control (data not shown). The amino acid sequences of P8 is Gag^378^MQRGNFRNQRKIVKC^392^, P9 is Gag^383^FRNQRKIVKCFNCGK^397^ and P12 is Gag^398^EGHIARNCRAPRKKG^412^ which proximity of the N-terminal NC domain covering the first conserved CCHC ZF1 and PRKK the basic residues which located between the ZF1 and ZF2.

**Figure 4:**
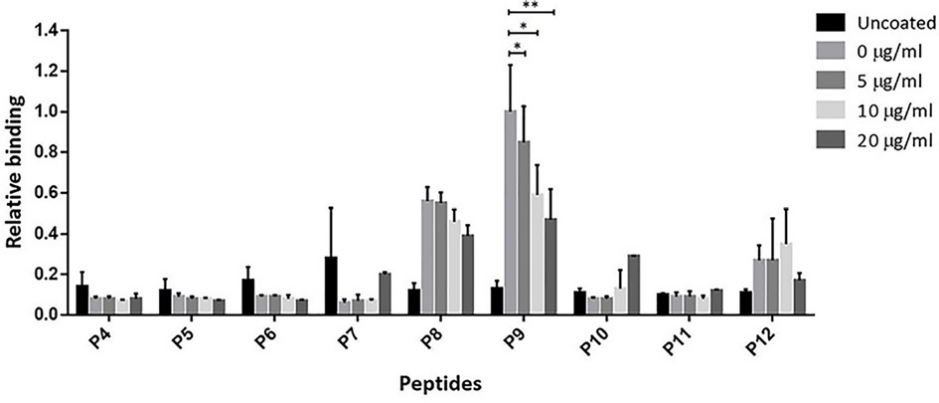
Competitive ELISA analysis for the specific binding of αRep9A8 and the short synthetic peptides The competitive reactions were reviewed by anti-His HPR and measured OD at 450 nm and the signals obtained from peptide coated well with no competitor in each set was attributed the 1-unit value. The relative binding activity was calculated using the following formula: [OD_with competitor_/OD_no competitor_] × 1. *y-axis*; the relative binding, *x-axis*; list of peptides coated wells (P4–P12). Data presented are from triplicate experiments (mean ± SD), analyzed using *t* test. *P<0.05 and **P<0.01.

The competitive ELISA was performed to confirm the binding activity of αRep9A8 and the synthetic peptides; P9, P8, and P12. The result obtained indicated that relative binding activities in the wells immobilized with P9 were significantly decreased when competing with CA_21_-SP1-NC at 5, 10, and 20 μg/ml in a dose-dependent response, respectively. A similar pattern was found in the wells coated with P8. Meanwhile, wells coated with other peptides did not give signals (Figure 4). The %inhibition of each concentration of competitor tested on P9 and P8 coated wells were calculated and showed in Table 2. In P8 immobilized well, competitor at 5, 10, and 20 μg/ml showed %inhibition at 2.28, 17.77, and 30.92%, respectively. The higher %inhibition was found in the reaction of P9 coated wells, at 5, 10, and 20 μg/ml of competitors, 15.09, 41.30, and 52.85% inhibition were shown. Therefore, the result suggested that αRep9A8 preferred to interact with the amino acid sequence located in P9 more than P8.

**Table 2:** Inhibition effect of GST-CA_21_-SP1-NC against candidate peptides

Peptide(s)	%Inhibition GST-CA_21_-SP1-NC (competitor)			
	0 μg/ml	5 μg/ml	10 μg/ml	20 μg/ml
P8	0%	2.28%	17.77%	30.92%
P9	0%	15.09%	41.30%	52.85%

### Binding kinetics of αRep9A8

**Figure 5:**
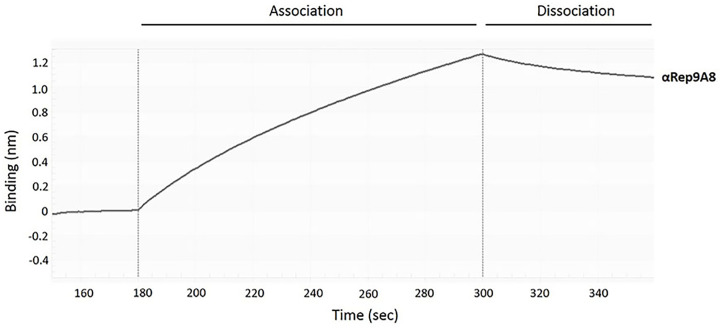
The binding kinetic of αRep9A8 Representative sensorgrams displaying the kinetic of the association and dissociation of αRep9A8 toward GST-CA_21_-SP1-NC. Binding curves were analyzed by the BLItz™ Pro 1.1 software.

To determine the specific binding of αRep9A8 toward target GST-CA_21_-SP1-NC, the kinetic bindings of the α repeat proteins were evaluated by BLI using the BLItz™ system. The sensorgrams that displayed the binding kinetic of αRep9A8 in the association step was approximately 1.2 nm (Figure 5). The equilibrium dissociation constant (*K*_D_) of the binding reaction between GST-CA_21_-SP1-NC and αRep9A8 was 224.9 ± 57.4 nM. Therefore, αRep9A8 exhibited a specific interaction to target GST-CA_21_-SP1-NC with a high binding affinity in the nanomolar range (Figure 5 and Table 3).

**Table 3:** Kinetic binding data of αRep9A8 determined by BLItz™ system (*n*=3)

Variant	*k*_on_ × 10^4^ (1/Ms)	*k*_off_ × 10^−3^ (1/s)	*K*_D_ (nM)
αRep9A8	1.5 ± 0.2	3.2 ± 0.4	224.9 ± 57.4

### Inhibition of HIV-1 proteolytic cleavage activity of αRep9A8

According to the binding site of αRep9A8 was mapped and the amino acid sequences of P8 took part in the protease cleavage site of SP1–NC junction, the first important cut site that HIV-PR performs proteolytic cleavage in the step of viral maturation. The experiment for determining of anti-PR activity was designed and analyzed by Western blotting. The result demonstrated that the reactions contain 20 µl of αRep9A8 at 2000, 1000, and 500 µg/ml could inhibit HIV-PRH_6_ cleavage as shown in lanes 1–3. No cleaved product GST-CA_21_-SP1, the 29 kDa was observed. Thicker protein bands of cleaved products were presented from lanes 4–6 which contain 20 µl of αRep9A8 at 250, 125, and 62.5 µg/ml, respectively. The biggest cleaved product band was clearly seen on lane 7 which has no αRep9A8 in the reaction mixture. No cleaved product band was seen in the control reaction which PI, lopinavir was added (lane 8). This suggested that αRep9A8 has could inhibit the proteolytic cleavage at SP1–NC junction.

## Discussion

Recently, therapeutic protein drugs have an impact on the overall healthcare industry and revolutionized alternative therapeutic approaches in various diseases [14]. Most of them are in a clinical trial such as recombinant protein for cancer therapies, immune disorders, infections, and others [21]. In the past few years, the number of repeat motif scaffold proteins has been established as molecular binder. The MP0112 (DARPin) (Molecular Partners/Allergen), an ankyrin repeat protein targeting retinal angiogenic disorders driven by VEGF-A entered Phase III clinical trials in 2015 [22]. Affibody specific to human epidermal growth factor receptor (HER2) [23] is being evaluated for imaging application in Phase I/II clinical trial [24]. PRS-110 (Anticalin), an artificial protein derived from human lipocalin directed against hepatocyte growth factor receptor (HGFR) has been investigated in the preclinical stage [25–27]. Previously, our group also reported that protein scaffolds including ankyrin repeat protein, zinc finger protein, and αRep protein could be used as intracellular antiviral molecules, and also demonstrated their therapeutic potential as HIV-1 inhibitors [17,28–31].

In the present work, we investigated the binding recognition site of the αRep protein scaffold called αRep9A8 which formerly reported as a negative interference of HIV-1 maturation [17]. The characteristic study of αRep9A8 is required to explore the molecular mechanism of this scaffold protein. The preliminary data of αRep9A8 epitope mapping was provided clues to their mode of action. Protein pull-down assay in our previous study showed that the αRep9A8 recognition site widely spanned along with the NC domain covering both ZF1 and ZF2 and possible to overlap the SP1–NC junction which is the HIV-1 PR cleavage site [17]. Indirect ELISA was used to specifically identify the binding site of αRep9A8 on the viral target protein, CA_21_-SP1-NC or HIV-1 Gag^358–412^. The short overlapping synthetic peptides, 15 amino acids long with 10 amino acids overlapped (P4–P12) were immobilized on the microtiter plate. The result demonstrated that αRep9A8 interacted with P9, P8, and P12 respectively. These peptide sequences were matched the N-terminal domain of NC covering the first conserved CCHC ZF1 and PRKK the basic residues located between the ZF1 and ZF2 (Figure 7). This finding correlated to our previous results from protein pull-down assay [17]. However, no binding signal of αRep9A8 to the P10 and P11 were detected in this experiment, it could be argued that short peptides 15 amino acids residues had altered its natural structure. In another way, it is possible to discuss that the binding site of αRep9A8 is a conformational epitope by which P10 and P11 were not required for its interaction. In addition, the competitive ELISA was performed using the viral target CA_21_-SP1-NC as a competitor to ensure the specific binding of αRep9A8 to its target. The dose-dependent relative binding and %inhibition obtained from different concentrations of competitors confirmed that αRep9A8 interacted with the amino acid residues within P8 and P9. Again, this verifies the specificity of the αRep9A8 to its partner. Moreover, the equilibrium dissociation constant of the αRep9A8 and viral target CA_21_-SP1-NC binding reaction was measured. The αRep9A8–CA_21_–SP1–NC complex demonstrated the binding affinity in the nanomolar range of values (*K*_D_ = 224.9 ± 57.4 nM). Even though the binding affinity was not as high as therapeutic antibodies which usually must have undergone the affinity maturation [32,33], among the families of scaffold repeat protein such as DARPins and αReps, the dissociation constant values are found from below nanomolar to the micromolar range [19,28,34,35]. Ank^GAG^1D4 and MA-CA formed a protein complex with a dissociation constant of *K*_D_ ∼1 μM [28]. In other studies, the dissociation constants of αRep protein targeting EGFP, bGFP-A–EGFP, and bGFP-C-EGFP complexes were also found to be in the nanomolar range, with *K*_D_ values of respectively 15 ± 4 nM [36] and 19 ± 12 nM [19].

**Figure 6:**
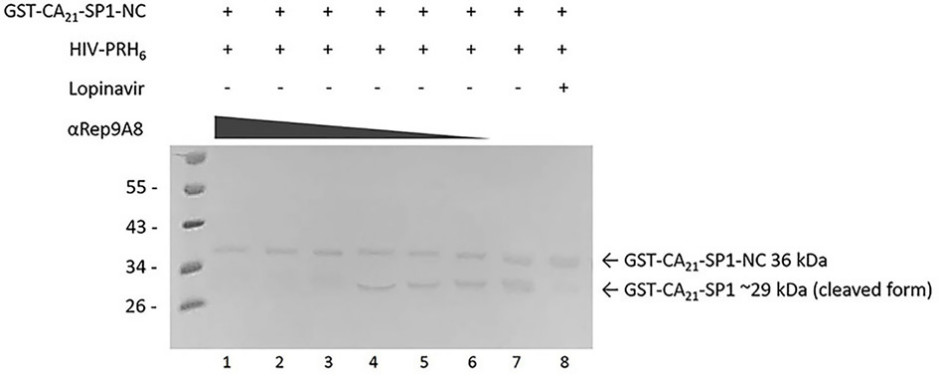
Determining of anti-protease activity of αRep9A8 The Western blotting analysis of the anti-protease activity of αRep9A8 at SP1–NC junction. The full-length viral target protein is GST-CA_21_-SP1-NC, the 36 kDa protein containing SP1–NC cleavage site. The cleaved product obtained from the proteolytic activity of PR at SP1–NC junction is GST-CA_21_-SP1, 29 kDa. In the reaction mixture, contains 20 µl of αRep9A8 at 2000, 1000, 500, 250, 125, 62.5, and 0 µg/ml (lanes 1–7), 20 µl of 5 mg/ml GST-CA_21_-SP1-NC, and 20 µl of 5 mg/ml HIV-PRH_6_. Lopinavir 1000 ng/ml, the PI was used as a control for preventing the PR cleavage activity (lane 8). The protein bands were reviewed by anti-GST monoclonal antibody and HRP-conjugated secondary antibody.

Binding of αRep9A8 at the N-terminus of NC resulting in the negative interference on HIV-1 proteolytic activity as shown in Figure 6. The Western blotting analysis demonstrated the anti-PR activity in a dose-dependent manner. Already effects of αRep9A8 were studied in human SupT1 cells challenged with laboratory strain HIV-1 NL4-3, we observed that αRep9A8 could interfere with the maturation process of the virus leading to impairment of viral infectivity and acquired a long-term resistance to HIV-1. This suggested that non-infectious viral production was a result of αRep9A8. The mechanism of action would be explained that binding of αRep9A8 to the viral target masking the SP1-NC PR cleavage site. As it bound to P9 stronger than P8, suggesting that αRep9A8 indirectly inhibit PR activity via steric hindrance rather than action by direct binding.

The viral maturation process is critical for the construction of the spherical virion of HIV-1 driven by the viral Gag proteins. The five proteolytic cleavage sites within Gag can be divided into three categories based on the rate of enzymatic processing; (i) the initial cleavage at the SP1–NC junction, the first and fastest one; (ii) sequent cleavages at NC-SP2-p6 and MA-CA, relative to the SP1-NC cleavage site, SP2-p6 is cleaved 9× slower and MA-CA is cleaved 14× slower; (iii) the final processing steps cleaves NC-SP2 at 350× slower and CA-SP1 at 400× slower. The late event especially the step for free SP1 from the CA plays a crucial role in the viral morphological maturation [1,2,37]. Regarding the sequential steps in the Gag processing, binding of αRep9A8 closed to the initial cleavage site could give a huge effect on the Gag processing efficiency and its downstream events. This correlated to the study of Ohishi et al., 2011, who reported that interfering with NC processing by PR from Gag precursor during the viral maturation step resulting in impairment of infectivity of the virus [8,38]. Bevirimat, one of a good example of an HIV-1 maturation inhibitor which pointed out that new drug options still needed to overcome the disadvantages of highly active antiretroviral drugs. This first-generation MI has a mechanism of action distinct from other antiretroviral drugs. Specifically blocked Gag processing at the rate-limiting step prevented the release of mature CA from CA-SP1 precursor. This event resulting in the production of non-infectious immature viral progenies [39,40]. This information guild us to the further study of αRep9A8 scaffold protein in terms of its application as a novel viral maturation inhibitor.

**Figure 7:**
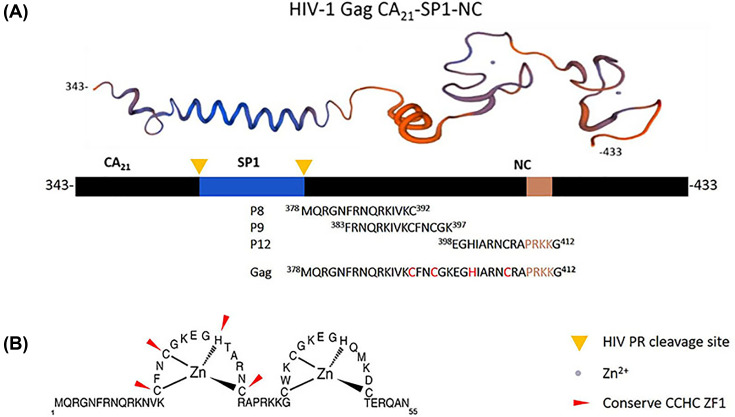
Amino acid sequence 3D structure, of the viral target protein (**A**) 3D structure of viral target protein, Gag^343–433^ (*top panel*), schematic representation of Gag^343–433^ with two indications of HIV PR cleavage site CA-SP1 and SP1-NC (*middle panel*), amino acid sequences of αRep9A8 binders and merged peptide sequence (*low panel*). (**B**) Fifty-five amino acid sequence of HIV NC showing the conserved CCHC zinc finger, ZF1 and ZF2, modified from Avilov et al. (2008) [45].

HIV-1 NC plays an important role in a large spectrum of viral activities. It is a key molecule for HIV-1 nucleic acid regulation. Specific and strong binding properties of NC to the ψ-encapsidation signal sequence on genomic RNA (gRNA) enabling the specific selection of the viral genome among a pull of cellular RNAs during particle assembly process [4,41,42]. The two conserve CCHC ZF motifs within NC are notably required to interact with the HIV-1 gRNA [4] (Figure 7B). Binding of αRep9A8 to its epitope on NC covering the ZF1 motif, Gag^378^MQRGNFRNQRKIVK**C**FN**C**GKEG**H**IARN**C**RAPRKKG^412^ (Figure 7A) could explain the anti-genome packaging in our previous publication [17]. The possibility to describe the mode of action of αRep9A8 would be the interaction to its target inhibited NC to bind to nucleic acid partners. It is also possible that αRep9A8 involves in the disturbing of zinc ion coordination [4]. Regarding the kinetic studies for the binding of PIs to wild type (WT) HIV-1 PR, they demonstrated the K_D_ values in a range, from 1.2 ± 0.3 nM (Saquinavir (SQV) to WT) to 0.41 ± 1.71 pM (DRV to WT) [43]. The second-generation MI (BMS-955179) functions by reversibly interacting with the CA-SP1 junction with *K*_D_ = 5.5 nM. It acquires similar anti-HIV-1 as αRep9A8 by potently blocking protease cleaving process [44]. Accordingly, declaring the αRep9A8 recognition site on CA_21_-SP1-NC and determining its binding kinetic is a prerequisite for further improvement of its function. Since the affinity of αRep9A8 is in the nanomolar range, affinity enhancement will be an ultimate goal.

## Conclusion

In the present study, we explored the specific binding site of thermoresistant scaffold protein, αRep9A8 using the portion of HIV-1 Gag polyprotein, CA_21_-SP1-NC as a template for designing nine overlapping synthetic short peptides. The results indicated that αRep9A8 bound to the N-terminal domain of the NC proximity to the SP1-NC initial cleavage site and across the conserved CCHC ZF1 of NC. The dissociation constant (*K*_D_) of the specific reaction of αRep9A8 with its target protein was in the nanomolar range. Besides, αRep9A8 was tested for the anti-HIV-1 PR activity. The αRep9A8 exhibits the negative effect on HIV-1 PR-mediated proteolytic cleavage by blocking the release of NC from the SP1-NC precursor. In summary, αRep9A8 bound to its target protein near the protease specific cut site and ZF1 motif resulting in disturbance of the viral maturation processes and decrease the efficiency of infectious viral progeny production.

## Abbreviations

BLI: bio-layer interferometry
CA: capsid
DARPin: designed ankyrin repeat protein
gDNA: genomic DNA
GST: glutathione-S-transferase
HRP: horseradish peroxidase
MA: matrix
NC: nucleocapsid
NCI: NC inhibitor
PI: protease inhibitor
PR: protease
PRH6: protease-His6 tag
SP1: spacer peptide 1
VEGF: vascular endothelial growth factor
ZF1: zinc finger 1
αRep: α-repeat
